# Predictable threats to public health through delaying universal access to innovative medicines for hepatitis C: a pharmaceutical standpoint

**DOI:** 10.1111/tmi.12784

**Published:** 2016-10-17

**Authors:** Raffaella Ravinetto, Anja De Weggheleire, Thomas P.C. Dorlo, Sven Francque, An Sokkab, Corinne Pouget, Bruno Meessen, Patricia Tabernero, Paul N. Newton, Lut Lynen

**Affiliations:** ^1^ Clinical Sciences DepartmentInstitute of Tropical MedicineAntwerpBelgium; ^2^ Clinical Pharmacology and PharmacotherapyKU LeuvenLeuvenBelgium; ^3^ Department Pharmaceutical BiosciencesUppsala UniversityUppsalaSweden; ^4^ Division of Pharmacoepidemiology & Clinical PharmacologyUtrecht UniversityUtrechtThe Netherlands; ^5^ University of AntwerpAntwerpBelgium; ^6^ Sihanouk Hospital Centre of HopePhnom PenhCambodia; ^7^ QUAMEDInstitute of Tropical MedicineAntwerpBelgium; ^8^ Public Health DepartmentInstitute of Tropical MedicineAntwerpBelgium; ^9^ INSERM U 1058MontpellierFrance; ^10^ Lao‐Oxford‐Mahosot Hospital‐Wellcome Trust Research UnitMahosot HospitalVientianeLao PDR; ^11^ Centre for Tropical Medicine & Global HealthNuffield Department of MedicineOxford UniversityOxfordUK; ^12^ Worldwide Antimalarial Resistance NetworkChurchill HospitalOxford UniversityOxfordUK; ^13^ London School of Hygiene and Tropical MedicineLondonUK

**Keywords:** essential medicines, counterfeit medicines, substandard medicines, access to medicines, quality of medicines, hepatitis C

## Introduction

Worldwide 80 million people are chronically infected with hepatitis C virus (HCV) 1, of whom 80% live in low‐ and middle‐income countries (LMICs). Analogous to the introduction of HAART in HIV/AIDS, new interferon‐free direct‐acting antiviral (DAA) treatment combinations have transformed the HCV treatment options 2, 3 with their potential to cure patients and stop the pandemic.

However, the high prices set by innovator manufacturers keep these novel medicines out of reach of most patients in LMICs 4, 5, because they prevent countries from integrating DAAs into treatment policies, and funding and implementing agencies from launching large‐scale treatment programmes. Prices have been pushed so high – sofosbuvir may cost up to US$ 85 000–110 000 per treatment course – that access has become a challenge even in high‐income countries, where in the majority, strict eligibility criteria are in place for allocating patients to these treatments 6, 7. The access strategies voluntarily put in place for LMICs by some pharmaceutical companies, although laudable, appear insufficient to achieve the goal of universal access 8, 9. Conversely, generic competition coupled to economies of scale could dramatically lower prices: a generic version of sofosbuvir is already available at less than US$ 500 per treatment course, and scaled‐up manufacturing is projected to achieve target prices of US$ 100–250 for a full generic treatment course within the next 15 years 10, 11, or potentially earlier. There is broad consensus among stakeholders that competition among quality‐assured generics is now urgently needed to enable the launch of large‐scale public treatment programmes and to change HCV treatment patterns worldwide 9.

An important step towards universal access to DAAs was the inclusion in 2015 of sofosbuvir, simeprevir, daclatasvir, dasabuvir, ledipasvir/sofosbuvir and ombitasvir/paritaprevir/ritonavir in the WHO Essential Medicines List (EML) 12, 13. This was accompanied by the expansion of the WHO Prequalification Programme to include medicines for hepatitis 14, 15. In the field of HIV/AIDS, the WHO Prequalification Programme has been a key element for the launch of large‐scale treatment programmes 16, by assessing the quality of generic antiretrovirals and building the capacity of medicines regulatory agencies (MRAs). But in that case, manufacturers of pre‐qualified generic products were benefiting from market rewards, as major stakeholders such as the Global Fund, UNITAID, PEPFAR etc. only purchase products pre‐qualified by the WHO or approved by SRAs 16, 17, thus creating an important market incentive for quality assurance. Such funding mechanisms do not exist for HCV today, thus there is no equivalent ‘market push’ for DAAs’ quality assurance. However, differently from HIV/AIDS, HCV treatment is not lifelong but for a limited time. Thus national governments might be less dependent on external funders and able to play this role themselves, if fairly‐priced, quality‐assured medicines were available.

## The challenge of ensuring pharmaceutical quality

Hopefully, the first generic DAAs will appear on the WHO Prequalification list very soon. But meanwhile, generic DAAs are already increasingly available in many LMICs, such as Cambodia, Bangladesh, Laos, Vietnam and India, where most often the full cost is borne by patients themselves.

The global pharmaceutical market is characterised by multiple standards 18: the quality of medicines is not uniform worldwide and largely depends on the level of income and regulation in the destination country 19, 20. Poor‐quality medicines are especially prevalent in insufficiently regulated contexts. Many reports from non‐HCV fields highlight the presence of either *substandard* medicines, that is genuine medicines that are produced by manufacturers authorised by an MRA but do not meet quality specifications, or *falsified* medicines, that is medicines deliberately and fraudulently mislabelled with respect to identity and/or source 21. Tragically, they are often detected *a posteriori,* when treatment failure or toxicity becomes evident.

For instance, miltefosine manufactured for the Bangladesh visceral leishmaniasis elimination programme did not contain its stated active ingredient, but the problem only surfaced after abnormally high numbers of treatment failures 22. In Pakistan, a substandard medicine caused by cross‐contamination at the manufacturing site was only discovered after it had killed 120 patients 23. More recently, falsified phenobarbital was discovered in Guinea Bissau after an unexpected increase in seizure frequency 24. Both substandard and falsified antimalarials have been increasingly documented over the last 15 years 25, 26, while profound increases in resistance rates to old antimalarials suggests that subtherapeutic treatment regimens exert covert drug pressure for drug resistance 27, 28.

While ‘repressive’ measures help prevent the circulation of falsified medicines by criminal organisations, such measures will do little to stem the circulation of substandard medicines, which requires *a priori* and sustained capacity strengthening of manufacturers, regulators and purchasers. The fight against poor‐quality medicines will only be effective if essential medicines are made accessible and affordable to all those in need 29.

Medicines for HCV are at high risk for these problems. In the absence of strong national programmes based on universal healthcare coverage and/or of major externally funded programs, most treatment providers in LMICs are private for profit: a market that is highly fragmented, difficult to monitor, and undemanding in terms of quality assurance. Some of the existing generic DAAs are or will be manufactured under the unilateral voluntary license agreement of Gilead for sofosbuvir and ledipasvir 30, which to our knowledge does not include mandatory WHO Prequalification or SRA registration 31. Bristol‐Myers Squibb's daclatasvir will be manufactured under voluntary license agreements negotiated through the Medicines Patent Pools (MPP) which requires WHO Prequalification or SRA approval 31, 32. Other innovative companies have not so‐far disclosed their strategies for HCV medicine access.

Other generic DAAs are produced by manufacturers not included in the licensing agreements, and/or in countries with MRAs that are not listed as stringent 17. For example, various DAA‐containing products are registered in Bangladesh, including fixed dose combinations 33; and many different sofosbuvir‐containing products are registered in Pakistan, either from local or Indian manufacturers 34. India, Brazil, Morocco and Egypt also have local production. Nevertheless, in a rapidly evolving environment, it is difficult to find clear and up‐to‐date information on all DAAs‐containing products currently manufactured and registered worldwide.

As long as DAAs quality is not certified either by WHO Prequalification or SRAs, policy makers and purchasers in LMICs are left without stringent guidance to untangle the web of DAAs generics’ quality. If poor‐quality products 21 were used at this stage of the pandemic, the consequences would be highly detrimental. For instance, medicines for which there is no proof of bioequivalence may not be therapeutically equivalent to the innovator: they could cause initially undetected therapeutic failure, and pose a clinical challenge when patients need retreatment (whether they be true non‐responders or not).

This is not a merely theoretical threat. Even if some patients have been successfully treated after purchasing generic HCV treatment online 35, there is also evidence of the circulation of falsified HCV medicines globally. The detection of falsified sofosbuvir and pegylated interferon and ribavirin has been highlighted by the lay press and in specialised fora since 2015 36, 37, 38, 39. The Consumer Protection Agency of Egypt issued in 2015 a ‘warning against Counterfeit Hepatitis C Sovaldi packs’ 40. In February 2016, the Egyptian company Pharco warned on its website about the presence in Myanmar of a falsified generic daclatasvir, falsely branded as a Pharco Corporation product (http://www.pharco.org/company-newsdetails.aspx?id=2447). WHO subsequently issued an alert of falsified versions of sofosbuvir 400 mg + ledipasvir 90 mg and of daclatasvir 60 mg in South‐East Asia 41. In March 2016, the Swiss Agency for Therapeutic Products, Swissmedic reported falsified Harvoni^®^ in Israel. The product, labelled as originating from India, was imported via a Swiss trading company and contained white instead of genuine orange coloured, diamond‐shaped tablets 42.

## Consequences of unregulated supply channels

The high prices of innovator products (‘one thousand dollar pills) and the ‘alternative’ routes to access treatment have quickly reached patients with HCV worldwide. Thus, access limitations have often driven interest in personal drug importation of the generic formulations outside the regulated channels 31.

A growing concern is the online purchase of generic DAAs, with or without medical prescription: an unregulated market that often constitutes the only ‘accessible’ opportunity for patients and private practitioners. It generally concerns the final product, but supply of raw DAAs’ active pharmaceutical ingredients to individual patients has been reported. At best, these ingredients are given by the patient to a local chemist, who will compound the final product into capsules; at worst, patients are left to self‐administer the raw substance. Online purchase of medicines is always risky if the supply chain is outside the control of SRAs. The purchase of active pharmaceutical ingredients that require compounding by a local chemist is even riskier. Products obtained by combining, mixing and otherwise altering drug ingredients are exempted of some good manufacturing practices procedures and marketing authorisation, but only for tailoring therapy to the needs of an individual patient in the absence of the corresponding speciality on the market 43. Treating HCV with medicines ‘assembled’ informally, outside the appropriate technical and regulatory framework, will expose patients to products that may be contaminated or of suboptimal therapeutic efficacy.

But desperate patients are willing to take the risk or are simply not aware of the dangers that this practice may entail. A patient writes in a blog: ‘I have had this Damocles sword swinging for over 20 years and it is such a relief to be actually doing something to rid myself of this unwelcomed virus’. Another one writes ‘…Fibroscan has jumped from 7 to 12 in under a year and my fatigue has skyrocketed. For me, time was running out and although the government may eventually come to the party with treatments I feel that they might rather want to spend the money on a nice bomb for the Air Force. I cannot afford to wait’. Only access to affordable quality‐assured medicines could discourage these people, in both rich and poor countries, from making use of potentially unsafe supply channels. Positive experience with generic DAAs purchased online has been recently reported in the frame of a clinical trial that included drug quality testing 35. However, the online purchase of medicines is fraught with difficulties, including falsification. The results from this experience may therefore not be replicable in real‐life settings, where the quality of DAAs cannot be controlled for every purchase and where ‘unscrupulous providers operating outside the import rules and regulations may prove to be a dangerous cost saving’ 31.

## Consequences of suboptimal use

Threats to individual and public health are not only linked to the risk of purchasing poor‐quality medicines, but also to DAAs’ suboptimal prescription and use, which is often triggered by poor availability and/or affordability. On the one hand, the intermittent availability of medicines in the private sector and the limited capacity of individuals to pay for them may heavily influence adherence to prescription patterns. Patients may be unable to complete a 12‐week treatment cycle or may opt to purchase only one drug of a treatment combination (as was frequently the case in the early days of HIV treatment).

On the other hand, patients living with chronic HCV often suffer from other important co‐infections or comorbidities. A patient treated within a (subsidised) HIV National Program may be concomitantly treated for HCV in the private sector, or in another country, and the lack of coordination between HIV and HCV caregivers is a major concern, because it will result in suboptimal management of the co‐infection and, in particular, little consideration for drug–drug interactions and possible cumulative toxicities 44. Lack of communication between HIV and HCV caregivers may also emerge in high‐income countries, for example when part of the care pathway is through consultations abroad: the promotion of HCV‐related medical tourism is increasingly reported 45 to obviate the strict allocation criteria in place 46.

The combined effects of poor‐quality medicines and of suboptimal medicines’ use (both triggered by the lack of affordable and quality‐assured treatment) contributed, in the case of malaria, to retrospectively detected increased morbidity/mortality 25, 26, 27. Even if the use of ineffective medicines will probably have a less immediately life‐threatening effect in HCV than in malaria, its effect on morbidity and avoidable human suffering remains a major public health impediment. In addition, patients treated with ineffective medicines will need retreatment, unnecessarily increasing the burden on health services and budgets.

## Recommendations to accelerate access to quality‐assured HCV treatment

If quality‐assured HCV treatments were available via the national programmes in the framework of universal care coverage, people living with HCV would not be pushed to seek treatment through non‐regulated supply chains, black markets or via a patchwork of parallel treatment‐seeking itineraries. Today, many patients are left with a terrible choice: stay away from treatment, opt for catastrophic expenses or look for alternative (often still expensive) sources of treatment that might expose them to suboptimal treatment outcomes or toxicity. These three scenarios are clearly at odds with the global ambition of universal health coverage 47 and the vision of combating hepatitis by 2030 (Sustainable Development Goal 3.3).

This is an urgent issue that needs to be addressed now. The WHO Prequalification or SRA registration should be an essential requirement for purchase by major stakeholders, such as national programmes, funding agencies and NGOs. Unrestrained competition among quality‐assured generics could, in particular, make the treatment scaling‐up feasible via the national programmes. Further delays in making quality‐assured, appropriately‐priced generics available to all those in need will create room for unregulated markets and push desperate patients to look for unsafe or suboptimal treatment, paving the way to treatment failure. Some non‐exhaustive, practical recommendations may be formulated to try to accelerate this process, by acting in parallel on the quality of generic DAAs and access to them:

- Market incentives should be in place to push the manufacturers of generic DAAs to get either WHO Prequalification or SRA registration. The WHO Prequalification or SRA registration should therefore be an essential requirement for purchase by all stakeholders, including national programmes, funding agencies, NGO and hopefully also the purchasers in the private sector. It should also be a condition for all DAAs’ voluntary licenses.
- The international community should invest in the reinforcement of the capacities of MRAs in LMICs, to allow them to develop stringent mechanisms for medicines registration. Meanwhile, MRAs in LMICs could put in place fast‐track registration processes for generic DAAs with WHO‐Prequalification or SRA registration.
- Innovator manufacturers should adopt efficient access policies to facilitate access in LMICs. For countries covered by voluntary licenses, transparent and patient‐centred mechanisms should be in place (managing voluntary licenses through the Medicines Patent Pool could facilitate this objective). For countries not covered by voluntary licenses, the application of all TRIPS flexibilities including compulsory licenses should pave the way for affordability and accessibility 9.
- Online distributors should be able to demonstrate that they are licensed by the competent authorities, and that the DAAs they supply have WHO‐Prequalification or SRA registration.
- Finally, to facilitate global access including in high‐income countries, those stringent regulatory authorities that grant the initial marketing authorisation could consider revising their current policies and engage in the evaluation of the pricing policies of new, innovative medicines 48.

The international community is now facing a choice: either wait and see whether poor‐quality DAAs and suboptimal therapies will spread further into the markets, and then react retrospectively; or take action now to prevent this from happening. If no action is taken – now – the lack of access to quality‐assured medicine will block the fight against a disease that could be cured and probably eliminated if adequate resources were mobilised.

## Conflict of interest

Lut Lynen received a research grant for ‘Hepatitis C treatment for HIV patients in resource‐poor countries: is a public health approach possible?’, funded by the Flemish Government's Department of Economy, Science & Innovation. Paul Newton is supported by the Wellcome Trust. The funders played no role in the content of the article or the decision to publish.
